# The Genetic Links to Anxiety and Depression (GLAD) Study: Online recruitment into the largest recontactable study of depression and anxiety

**DOI:** 10.1016/j.brat.2019.103503

**Published:** 2019-12

**Authors:** Molly R. Davies, Gursharan Kalsi, Chérie Armour, Ian R. Jones, Andrew M. McIntosh, Daniel J. Smith, James T.R. Walters, John R. Bradley, Nathalie Kingston, Sofie Ashford, Ioana Beange, Anamaria Brailean, Anthony J. Cleare, Jonathan R.I. Coleman, Charles J. Curtis, Susannah C.B. Curzons, Katrina A.S. Davis, Le Roy C. Dowey, Victor A. Gault, Kimberley A. Goldsmith, Megan Hammond Bennett, Yoriko Hirose, Matthew Hotopf, Christopher Hübel, Carola Kanz, Jennifer Leng, Donald M. Lyall, Bethany D. Mason, Monika McAtarsney-Kovacs, Dina Monssen, Alexei Moulton, Nigel Ovington, Elisavet Palaiologou, Carmine M. Pariante, Shivani Parikh, Alicia J. Peel, Ruth K. Price, Katharine A. Rimes, Henry C. Rogers, Jennifer Sambrook, Megan Skelton, Anna Spaul, Eddy L.A. Suarez, Bronte L. Sykes, Keith G. Thomas, Allan H. Young, Evangelos Vassos, David Veale, Katie M. White, Janet Wingrove, NIHR BioResource consortium, Thalia C. Eley, Gerome Breen

**Affiliations:** a Institute of Psychiatry, Psychology and Neuroscience, King's College London, Denmark Hill, Camberwell, London, UK; b UK National Institute for Health Research (NIHR) Biomedical Research Centre, South London and Maudsley Hospital, London, UK; c School of Psychology, Queens University Belfast (QUB), Belfast, Northern Ireland, UK; d MRC Centre for Neuropsychiatric Genetics and Genomics, Neuroscience and Mental Health Research Institute, Cardiff University, Cardiff, UK; e Division of Psychiatry, Centre for Clinical Brain Sciences, University of Edinburgh, Edinurgh, UK; f Institute of Health and Wellbeing, University of Glasgow, Glasgow, UK; g NIHR BioResource, Cambridge University Hospitals NHS Foundation, Cambridge Biomedical Campus, Cambridge, UK; h Department of Haematology, University of Cambridge, Cambridge Biomedical Campus, Cambridge, UK; i Department of Public Health and Primary Care, University of Cambridge, Cambridge, UK; j South London and Maudsley NHS Foundation Trust, Bethlem Royal Hospital, Monks Orchard Road, Beckenham, Kent, UK; k School of Biomedical Sciences, Ulster University, Coleraine Campus, Northern Ireland, UK; l GreenLight Pharmaceuticals Limited, Unit 2, Block E, Nutgrove Office Park, Dublin 14, Ireland; m Department of Medical Epidemiology and Biostatistics, Karolinska Institutet, Stockholm, Sweden

**Keywords:** Anxiety, Depression, Behavior genetics, Psychiatric genetics, Data sharing, Life events, GWAS, genome-wide association study, SNP, single nucleotide polymorphism, GLAD, Genetic Links to Anxiety and Depression, NIHR, National Institute for Health Research, IAPT, Improving Access to Psychological Therapies, SLaM, South London and Maudsley, NHS, National Health Service, SURE, Service User Research Enterprise, FAST-R, Feasibility and Acceptability Support Team for Researchers, PR, public relations, SOPs, standard operating procedures, CRN, Clinical Research Networks, GP, general practitioner, CCG, Clinical Commissioning Group, MDD, Major depressive disorder, GAD, Generalised anxiety disorder

## Abstract

**Background:**

Anxiety and depression are common, debilitating and costly. These disorders are influenced by multiple risk factors, from genes to psychological vulnerabilities and environmental stressors, but research is hampered by a lack of sufficiently large comprehensive studies. We are recruiting 40,000 individuals with lifetime depression or anxiety and broad assessment of risks to facilitate future research.

**Methods:**

The Genetic Links to Anxiety and Depression (GLAD) Study (www.gladstudy.org.uk) recruits individuals with depression or anxiety into the NIHR Mental Health BioResource. Participants invited to join the study (via media campaigns) provide demographic, environmental and genetic data, and consent for medical record linkage and recontact.

**Results:**

Online recruitment was effective; 42,531 participants consented and 27,776 completed the questionnaire by end of July 2019. Participants’ questionnaire data identified very high rates of recurrent depression, severe anxiety, and comorbidity. Participants reported high rates of treatment receipt. The age profile of the sample is biased toward young adults, with higher recruitment of females and the more educated, especially at younger ages.

**Discussion:**

This paper describes the study methodology and descriptive data for GLAD, which represents a large, recontactable resource that will enable future research into risks, outcomes, and treatment for anxiety and depression.

## Highlights

• Online recruitment of 40,000 individuals with lifetime depression or anxiety.
• Detailed online phenotyping combined with genetic and clinical data.
• The study sample is severe, highly comorbid, with chronic psychopathology.
• The study protocol enables recall of participants for future research and trials.

## 1 Introduction

Anxiety and depression are the most common psychiatric disorders worldwide, with a lifetime prevalence of at least 30% (Bandelow & Michaelis, 2015; Kessler et al., 2005). Both are highly comorbid with each other and with other psychiatric disorders (Mineka, Watson, & Clark, 1998), and account for 10% of all years lived with disability (World Health Organisation, 2017). The World Health Organisation now considers depression to be the number one disorder by burden of disease (World Health Organisation, 2017). Many risk factors for depression and anxiety are shared, including psychological (e.g. cognitive biases (Michl, McLaughlin, Shepherd, & Nolen-Hoeksema, 2013; Naragon-Gainey, 2010)), environmental (e.g. stressful life events (Michl et al., 2013)), and genetic influences (which are ∼60–100% shared (Purves et al., 2017; Waszczuk, Zavos, Gregory, & Eley, 2014)). These findings on aetiology have been accompanied by an increasing evidence base of effective treatments, especially psychological (talking) therapies (Deacon & Abramowitz, 2004; Dobson, 1989; Hofmann & Smits, 2008). Nevertheless, despite advancements in psychological therapies as well as medication, clinicians are unable to predict which treatment will work for whom. This means that the choice of first and subsequent treatments currently progresses by trial and error at the cost of prolonged disability, reduced hope of success/engagement, and increased risk of adverse events (Perlis, 2016; Rush et al., 2006).

Decades of work estimate twin study heritability of both anxiety and depression at ∼30–40% (Sullivan, Neale, & Kendler, 2000), rising to ∼60–70% for the recurrent forms of these disorders across several years (Kendler, Neale, Kessler, Heath, & Eaves, 1993). Recent UK Biobank analyses confirm that common genetic variants (single nucleotide polymorphisms, or “SNPs”) account for a smaller but still significant ∼15–30% of variation (“SNP-heritability”) in lifetime anxiety (Purves et al., 2017). Depression has a SNP-heritability of ∼12–14% and shows substantial genetic overlap with anxiety (Purves et al., 2017). Recent genome-wide association studies (GWAS) have identified 102 genetic variants for depression and closely related phenotypes (Howard et al., 2019) and 5 genetic variants for anxiety (Purves et al., 2017), indicating that we are now entering the era where it will be possible to finally discover new biology for both disorders. In addition, these genetic advances suggest that it may be time to include genetic risk factors in research aimed at developing new treatments or predicting which therapies will work best for each patient (Breen et al., 2016; Gaspar et al., 2018; Keers et al., 2016; Lewis and Vassos, 2017).

Both GWAS and sequencing studies have shown that there are few, if any, individual genetic variants with a large effect size. Instead, the overall heritability is made of the effect of many (probably thousands) genetic variants that individually have small effects. This type of genetic architecture is now commonly referred to as being polygenic. As such, almost all disorders affecting >0.5% of the population that show moderate to high heritability are now referred to as polygenic disorders. The use of genome-wide methodology has also confirmed that psychiatric genetics has moved beyond its replication crisis, as its results have proven much more replicable than those of candidate gene studies (Border et al., 2019).

Despite the broad range of established risk factors (Burcusa & Iacono, 2007; Craske et al., 2017; Fliege, Lee, Grimm, & Klapp, 2009), it is unclear how different types of influences combine to increase risk and influence treatment response. In particular, clinical/psychological (e.g. comorbidities), demographic (e.g. age), environmental (e.g. life events), and genetic influences have been studied largely independently, despite their evident interplay (Kendler, Gardner, & Prescott, 2002). As such, more multidisciplinary research is needed. However, efforts to better understand the aetiology of these disorders require large sample sizes and detailed information on symptom presentation. Many studies exist worldwide with either the scale of participants or the thorough phenotyping needed, but few have both. In response to this, the Genetic Links to Anxiety and Depression (GLAD) Study was developed to recruit >40,000 individuals into the newly established National Institute for Health Research (NIHR) Mental Health BioResource. Our sample size was based on the available funding and our aim to be one of the largest single studies (as opposed to previous meta-analyses) of individuals with a lifetime experience of clinical depression and anxiety, providing sufficient power for discovery analyses and for recall studies.

The NIHR Mental Health BioResource is an integral part of the overall NIHR BioResource for Translational Research (https://bioresource.nihr.ac.uk/), which has previously recruited approximately 100,000 individuals. However, a large majority of these volunteers were either healthy controls or have a physical health condition with no reported mental health disorder. This gap led to the creation of the NIHR Mental Health Bioresource. GLAD is its first project and has the specific goal of recruiting a large number of participants with anxiety and depression to facilitate future recall and secondary data analysis studies. Recall studies would involve recontacting participants to take part in further research, such as clinical trials aimed at developing better therapies and interventions for anxiety and depression. The GLAD Study will also directly explore both genetic and environmental risk factors for depression and anxiety disorders, including the potential of polygenic risk scores (Purcell et al., 2009) created from analyses of related phenotypes to predict response to treatment and prognosis. GLAD aims to facilitate studies which need to combine genetic, phenotypic, and environmental exposure data by creating a large, homogeneously phenotyped cohort of individuals with depression or anxiety with all these types of data.

## 2 Methods

### 2.1 Pre-launch preparation

**Fig. 1:**
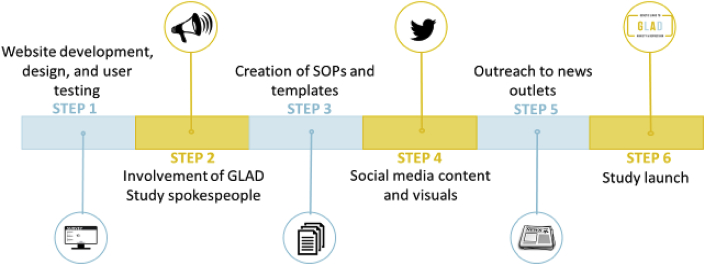
Steps to prepare for launch of the GLAD Study.

A focused and intense social media campaign was utilised to inform the public about the GLAD Study. We hoped that individuals with anxiety and depression would be encouraged to join given the online methodology, allowing them to respond to the questionnaires in a convenient time and location. The timeline leading up to the media launch is outlined in Fig. 1. Experts in the field, patients, and individuals with lived experience of depression or anxiety were consulted at each stage of study development. The study team also consulted regularly with collaborators from the Australian Genetics of Depression Study who provided guidance and advice based on their experience of successfully implementing a similar study design in Australia (Byrne et al., 2019).

#### 2.1.1 Website development, design, and user testing

The website was developed in collaboration with a local company (Mindwave Ventures) in various stages. During the early stages of development and the design phase, we conducted user testing on the website by contacting individuals with lived experience through various routes. The first user testers were patient volunteers within the Improving Access to Psychological Therapies (IAPT) service at South London and Maudsley (SLaM) National Health Service (NHS) Foundation Trust. The second group of volunteers were members of the King's College London Service User Research Enterprise (SURE) and the Feasibility and Acceptability Support Team for Researchers (FAST-R). Third were volunteer individuals with lived experience who were actively involved with the charity Mind. Finally, staff from the charities Mind, MQ Mental Health, and the Mental Health Foundation gave feedback. All individuals provided input on the website content and usability, appearance, and study information and all volunteers (but not charity staff) were compensated £10 for their time. All feedback was reviewed by the study team and the lead investigators and incorporated into the final version of the website.

#### 2.1.2 GLAD Study spokespeople

A wide range of charities, professional bodies, companies, influencers (defined as individuals with a large number of followers on social media), and individuals with lived experience were identified based on previous advocacy, involvement, or openness of mental health and treatment. These individuals and organisations were approached by the public relations (PR) company (Four Health Communications), the study team, and/or the co-investigators to introduce the study and invite them to collaborate.

We received support from many UK charities including Mind, MQ, Mental Health Foundation, Anxiety UK, OCD Action, Bipolar UK, HERO, No Panic, the Charlie Waller Memorial Trust, BDD Foundation, Maternal OCD, Rethink Mental Illness, SANE, MHFA England, Universities UK, and UK Youth. We also received significant support from two UK professional bodies for psychologists and psychiatrists: the British Psychological Society and the Royal College of Psychiatrists. Finally, three organisations (Royal Mail, Priory Group, and Barnardo's) agreed to circulate study information internally to their employees, and Priory Group additionally shared study information on social media.

Many of the charities, professional bodies, and influencers provided a quote about the GLAD Study to be shared with news outlets and on social media. Other forms of support from charities or professional bodies included circulating study information in member newsletters or magazines, publishing study information on their websites, and sharing the study on social media (http://wke.lt/w/s/Ymfhz). Influencers helped to promote the study on their social media channels or blogs. Individuals with lived experience allowed the GLAD Study team to share their stories on social media and news outlets. Of utmost importance, these individuals were available for interviews with broadcast and newspaper agencies and gave a personal voice to the campaign. Spokespeople who became actively involved in the publicity and circulation of the GLAD Study in the media and/or online were vital to the success of the campaign.

#### 2.1.3 Standard operating procedures and templates

We created a variety of standard operating procedures (SOPs) and response templates to prepare for the logistical and administrative aspects of study launch and management. These included SOPs for saliva sample kit preparation and posting, website management, data protection, and participant contact. We established guides and SOPs for both email and social media responses to participant questions, concerns, and expressions of distress. These guidelines were reviewed by clinicians and by the Mental Health Foundation to ensure responses were appropriate, straightforward, and clear.

#### 2.1.4 Social media content and visuals

Social media content and visuals were designed with the aid of the PR company (which had experience of working on NHS projects), an independent videographer, and an animation company. These included a range of infographics and short videos, each designed to be informative and provide basic details for the public about the aims of GLAD and how to join the project. The campaign was targeted to a younger demographic (age 16–30) to enable more prolonged collection of longitudinal data and reflect the young age of onset of depression and anxiety disorders (Bandelow & Michaelis, 2015; Christie et al., 1988; Kessler et al., 2005).

**Fig. 2:**
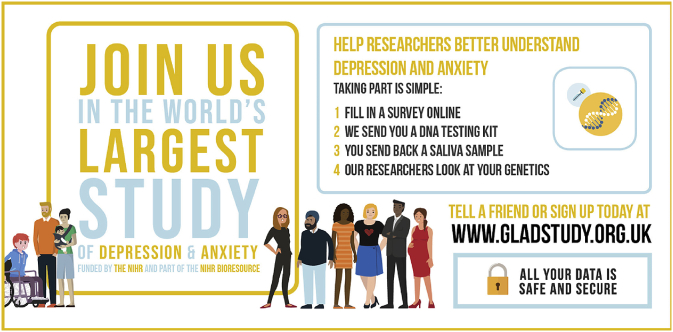
GLAD Study infographic shared on Twitter Example of the infographic shared on Twitter to promote the GLAD study and explain the key steps involved in signing up.

The infographics (Fig. 2) emphasised the simplicity and ease of taking part. The consent animation (https://youtu.be.com/SAE0yVNvWaA) outlined key elements of the consent process to provide a simple and clear way to learn about the study, consistent with guidance from the Health Research Authority regarding multimedia use as a patient-friendly method of delivering vital consent information (Health Research Authority, 2017). The consent animation was also included with the website version of the information sheet. The second video was a short film (https://youtu.be/wzgvS8gU2Ss) which outlined the study sign-up steps in a real-life, relatable way. Finally, the animation (https://youtu.be.com/HUI5eFXevvk) was an engaging ‘call to action’ to join the community of volunteers participating in the GLAD Study.

We created a six-week social media schedule for planned posts across Facebook, Instagram, and Twitter platforms which included both organic and promoted content. Most of the posts were accompanied by a video, infographic, or other imagery developed by the study team.

#### 2.1.5 Outreach to news outlets

The PR company circulated the press release and study information to news and broadcast agencies across England, and later across the rest of the UK. Interested parties organised interviews with the study investigators and/or influencers, charity or professional body representatives, and individuals with lived experience. The press releases and broadcasts were embargoed until the launch date to prevent pre-emptive publicity and provide uniform, widespread coverage across different media.

### 2.2 Recruitment

#### 2.2.1 Media campaign: study launch

The PR company helped us to organise a widespread national media campaign which included traditional media (TV, radio, newspaper) and social media (Twitter, Facebook, Instagram) outreach during the study launch. Examples from the campaign can be viewed at this link: http://wke.lt/w/s/Ymfhz. In the first 24 h, 8004 participants had registered to the website, demonstrating the effectiveness of this strategy. The prepared social media schedule helped to maintain our recruitment numbers over the six-week period. Between 300–1500 participants signed up to the study daily, with numbers fluctuating based on campaign expenditure and social media support from influencers and organisations.

Five months after the initial launch, the GLAD Study opened in Northern Ireland, Scotland, and Wales, in collaboration with researchers at Ulster University,^2^ the University of Edinburgh, and Cardiff University. The press offices of the respective universities organised media outreach to local and national news outlets, and the PR company assisted in targeted social media advertisements for the various countries.

#### 2.2.2 Clinical recruitment

In the 10 months following the launch, many NHS organisations in England contacted us interested in supporting the GLAD Study. As of July 2019, these included 12 Clinical Research Networks (CRN), 7 family doctor/general practitioner (GP) practices, 96 Trusts, 1 Clinical Commissioning Group (CCG), and 2 GP Federations. Sites were classified in 2 different categories with varying levels of involvement: 1) advertising sites which displayed study posters and leaflets in clinics and waiting areas, and 2) recruiting sites which conducted mail-outs, approached patients in the clinic or over the phone, and assisted patients through the study sign-up process.

#### 2.2.3 Sign-up process

**Fig. 3:**
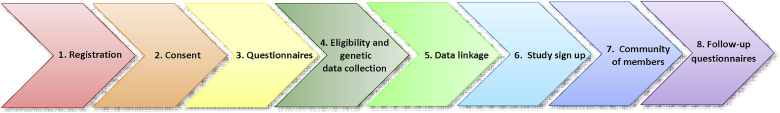
Stages of participant sign-up and involvement in the GLAD Study.

The sign-up process for enrolment in GLAD is given in Fig. 3. Personal information and phenotypic data are collected entirely online through the GLAD Study website (www.gladstudy.org.uk). Participants register on the website with their name, email address, phone number, date of birth, sex, and gender. They are then able to read the information sheet and provide consent. As part of the consenting process participants agree to long-term storage of their sample, requests to complete follow-up questionnaires, anonymised data sharing, recontact for future research studies based on their phenotype/genotype information, and access to their full medical and health related records. Following consent, participants complete the sign-up questionnaire to assess their eligibility. Eligibility criteria are restricted to participants that meet DSM-5 criteria for MDD or any anxiety disorder, based on responses to screening measures in our online sign-up questionnaire. More information about the measures in the sign-up questionnaire are included below. Eligible participants are then sent an Isohelix saliva DNA sample kit. Saliva samples are sent via Freepost to the NIHR National Biosample Centre in Milton Keynes for processing and storage. Once participants have returned their saliva sample, they become full members of the GLAD Study (and of the NIHR BioResource) and are eligible to take part in future studies.

Email reminders are sent to participants who do not complete their sign-up steps. Up to four automated reminder emails are sent through the website up to six months post starting sign-up. Once online sign-up steps are completed, a maximum of four reminder emails are sent to participants regarding the saliva samples up to six months from the date the kit was sent. We conduct additional phone call or text message reminders to participants who have not returned their saliva kits within three months of signing-up.

Clinical data will be linked to genotype and phenotype data to provide additional information about participants’ medical history, diagnoses, and treatments relevant to current and future research projects. Eligibility for collaborating studies can then be assessed utilising all phenotypic, genetic, and clinical data. Members may then be contacted up to four times per year to take part in future studies, either through the website or by the GLAD Study or NIHR BioResource teams, with access granted via established NIHR BioResource access protocols (see below for further detail). As part of the community of members, participants will also have access to useful information and links on the “Useful Links” page of the study website. Members will also be invited to take part in follow-up questionnaires to provide longitudinal data on their symptoms.

### 2.3 Measures

#### 2.3.1 Sign-up questionnaire

The sign-up questionnaire was designed to assess core demographic, mental and physical health, comorbidities, and personal information as well as detailed psychological and behavioural phenotyping relevant to anxiety and depression (for full measure names see Table 1). We included lifetime measures of major depressive disorder (MDD), atypical depression, and generalised anxiety disorder (GAD), adapted from CIDI-SF (Patten, Brandon-Christie, Devji, & Sedmak, 2000) and ICD-11 checklists (Kogan et al., 2016), supplemented with items enabling lifetime assessment of DSM-5 specific phobia, social phobia, panic disorder and agoraphobia. These items were adapted from the Australian Genetics of Depression Study questionnaire (Byrne et al., 2019).

**Table 1:** GLAD Study sign-up questionnaire measures.

Assessment/Topic	Purpose	Source
Lifetime depression	Lifetime assessment of major depressive disorder	Adapted Composite International Diagnostic Interview – Short Form (CIDI-SF)
Lifetime atypical depression	Lifetime assessment of atypical depression	Adapted DSM-5/ICD-11 checklists
Lifetime anxiety	Lifetime assessment of generalised anxiety disorder	Adapted Composite International Diagnostic Interview – Short Form (CIDI-SF)
Lifetime specific phobia	Lifetime assessment of specific phobia	Adapted DSM-5/ICD-11 checklists
Lifetime social phobia	Lifetime assessment of social phobia	Adapted DSM-5/ICD-11 checklists
Lifetime panic disorder	Lifetime assessment of panic disorder	Adapted DSM-5/ICD-11 checklists
Lifetime agoraphobia	Lifetime assessment of agoraphobia	Adapted DSM-5/ICD-11 checklists
Current depressive symptoms	Assess severity and presence/absence of current depression.	Patient Health Questionnaire 9-items (PHQ-9)
Treatment-resistant depression	Assess treatments during most recent or current depressive episode to screen for treatment-resistant	Maudsley Staging Model of Treatment Resistance, Modified Self-Report (MSM-SR)
Possible mania/hypomania	Screen participants for possible episodes of hypomania or mania	The Mood Disorder Questionnaire (MDQ)
Current general anxiety symptoms	Assess the severity and presence/absence of current anxiety	Generalised Anxiety Disorder 7-item scale (GAD-7)
Current post-traumatic stress disorder symptoms	Screen for presence and severity of PTSD symptoms.	Post-traumatic stress disorder checklist, civilian version 6-items (PCL-6)
Alcohol use	Screen for alcohol dependence in the past year	Alcohol Use Disorders Identification Test (AUDIT)
Psychotic symptoms	Assess lifetime psychotic experiences	Adapted CIDI
Personality disorders	Assess the severity of personality disorders.	Standardized Assessment of Severity of Personality Disorder 9-items (SASPD)
Work and social adjustment	Assess functional impairment due to psychopathology	Work and Social Adjustment Scale (WSAS)
Subjective wellbeing	Assesses subjective well-being, specifically positive emotion and meaning	Two hedonic (‘positive emotion’) questions and a eudaimonic (‘meaning’) question from WHO-Quality of Life (WHOQOL)
Adversities in childhood	Identify presence/absence of childhood abuse and neglect	Childhood Trauma Screener short version (CTS-5)
Adult domestic abuse and catastrophic trauma	Identify victims of adult domestic abuse.	A 5-item domestic abuse screen developed for the UK Biobank Mental Health Questionnaire, adapted from the national crime survey and CTS-5, and 5 items on catastrophic trauma

We also assessed current depressive symptoms (PHQ-9 (Kroenke, Spitzer, & Williams, 2001)), and those with a score of five or above on the PHQ 9 (indicating a current episode of depression) were asked additional questions related to current treatment-resistant depression (MSM-SR (Fekadu, Donocik, & Cleare, 2018)). Other measures of current psychopathology included possible mania/hypomania (MDQ (Hirschfeld, 2002)), current general anxiety symptoms (GAD-7 (Spitzer, KroenkeWilliams, & Löwe, 2006)), post-traumatic stress disorder symptoms (PCL-6 (Lang et al., 2012)), alcohol use (AUDIT-C (Saunders, Aasland, Babor, de La Fuente, & Grant, 1993)), psychotic symptoms (adapted CIDI (McGrath et al., 2015)), personality disorder symptoms (SAS-PD (Olajide et al., 2018)), and work and social adjustment (WSAS (Marks, 1986)). Additional measures were included to assess subjective well-being (WHOQOL Group, 1994), recent adverse life events, five childhood trauma items (Glaesmer et al., 2013) representing the five subscales of the full Childhood Trauma Questionnaire (Pennebaker & Susman, 2013), domestic violence (Davis et al., 2018), and catastrophic trauma (Davis et al., 2018).

To facilitate future meta-analyses with other cohorts, measures were selected when possible to align with the UK Biobank Mental Health Questionnaire (MHQ) (Davis et al., 2018). Participants were also asked to report if they have taken part in the UK Biobank. Some aspects of the UK Biobank MHQ were not selected for the sign-up questionnaire in order to make space for additional measures more relevant here. Furthermore, detailed questions on self-harm and suicide were not asked during sign-up due to concerns from the study team and the SLaM Research and Development (R&D) department of insufficient clinical support in the case of reported adverse events, although the single suicidal ideation item in the PHQ-9 was retained as this measure is validated and widely used.

#### 2.3.2 Optional questionnaires

Once participants completed the sign-up questionnaire, they were invited to take part in additional, optional questionnaires (SM 1). Optional questionnaires to assess a wider number of psychiatric phenotypes and symptoms included measures of fear (Fear Survey Schedule (Wolpe & Lang, 1964)), drug use (DUDIT (Berman, Bergman, Palmstierna, & Schlyter, 2003)), obsessive-compulsive disorder (OCI-R (Foa et al., 2002)), post-traumatic stress disorder (PCL-5 (Weathers et al., 2013)), trauma (Kopenen, personal communication), postnatal depression (EPDS (Cox, Holden, & Sagovsky, 1987)), body dysmorphic disorder (DCQ (Mancuso, Knoesen, & Castle, 2010)), eating disorders (Allison, Stunkard, & Thier, 2004; Herman et al., 2016; Hildebrandt, Jim Langenbucher, & Schlundt, 2004; Thornton et al., 2018), and vomit phobia (SPOVI (Veale, Ellison, Mark, & BoschenCostaWhelanMuccioHenry, 2013)).

Other optional projects relate to lifestyle and personal history, asking detailed questions on participants’ experience of healthcare, life events (Weathers et al., 2013), work and sleep, general health and lifestyle, gambling, and headaches and migraines (Byrne et al., 2019). Optional questionnaires were made available to all participants, except those on vomit phobia and postnatal depression which were only offered to participants based on responses to screening questions in the sign-up questionnaire. Additional follow-up questionnaires will be sent to participants annually to provide longitudinal data on symptoms and outcomes.

### 2.4 Genotyping

All GLAD samples will be genotyped as part of core NIHR BioResource funding. For genotyping we are using the UK Biobank v2 Axiom array, consisting of >850,000 genetic variants (Bycroft et al., 2018), designed to give optimal information about other correlated genetic variants. This careful design means that imputation of the data with large whole genome sequencing reference datasets will yield >10 million common genetic variants per individual. We use Affymetrix software and the UK Biobank pipeline software to assign genotypes and perform standard quality control measures in PLINK (Chang et al., 2015) or equivalent software packages and R (Team, 2015). Analyses will be conducted in PLINK in the first instance.

### Ethical approval

The GLAD Study was approved by the London - Fulham Research Ethics Committee on 21st August 2018 (REC reference: 18/LO/1218) following a full review by the committee. The NIHR BioResource has been approved as a Research Tissue Bank by the East of England - Cambridge Central Committee (REC reference: 17/EE/0025). Prior to submission for ethical approval, this research was reviewed by a team with experience of mental health problems and their carers who have been specially trained to advise on research proposals and documentation through the Feasibility and Acceptability Support Team for Researchers (FAST-R): a free, confidential service in England provided by the NIHR Maudsley Biomedical Research Centre via King's College London and South London and Maudsley NHS Foundation Trust.

### 2.5 Analysis

#### 2.5.1 Genetics and combined predictors of anxiety and depression

The GLAD data can be used alongside UK Biobank for meta-analyses, with UK Biobank participants and/or other NIHR Bioresource participants as healthy controls, to maximise statistical power. One of our primary genomic aims is to utilise polygenic scores created from very large genome-wide analyses of related traits (e.g. anxiety) as potential predictors of depression, anxiety, and treatment response in our sample. There are a number of well-powered polygenic scores now available in the field not only for psychiatric traits but also intelligence and its proxies (Selzam et al., 2017) and other relevant predictors. We aim to combine these polygenic scores with significant clinical predictors to produce a combined clinical/genetic risk index.

We envision the GLAD sample to also be incorporated either in large genome-wide association studies, contributing to meta-analyses, or in clinical trials, for example researching genetic, epidemiological or social risk factors of anxiety and depression.

### 2.6 Future research and collaborations

Researchers wishing to access GLAD Study participants or data are invited to submit a data and sample access request to the NIHR BioResource to request a collaboration, following the procedures outlined in the access request protocol (SM 2). Applications will be reviewed by the NIHR Steering Committee to assess the study aims and to check ethical approvals and protocols. Collaborations can range from sharing of anonymised data or samples to recontact of participants for additional studies, including experimental medicine studies and clinical trials. Eligibility for future research can be targeted to specific genotypes and/or phenotypes of interest.

## 3 Results

### 3.1 Sample descriptors

Recruitment of the GLAD Study is ongoing. All results that follow are from participants recruited before 25 July 2019. As of this date, 42,531 participants had consented to the GLAD Study, with approximately 35% drop-off rate at each stage of the sign-up process. This resulted in 27,991 (65.8%) having completed the questionnaire, with 27,776 (99.2%) screened as eligible and sent a saliva kit. Of those participants which were sent a kit, 18,663 (67.2%) have returned a saliva sample thus far.

**Fig. 4:**
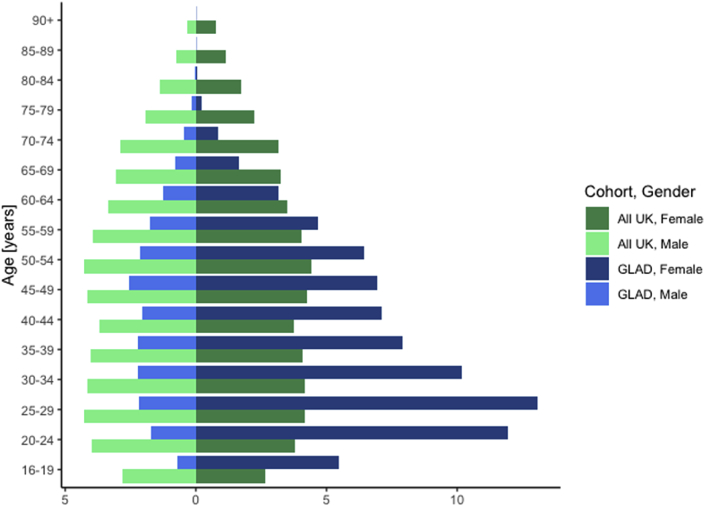
Distribution of sex and age in the GLAD sample compared to the UK population Comparison of age and sex characteristics between the GLAD sample and the general population of the UK as measured in the 2011 census (Office for National Statistics, National Records of Scotland, and Northern Ireland Statistics and Research Agency, 2016). Proportions of males in each age group are displayed on the left side of the graph, with female proportions on the right. Percentages represent the proportion out of the respective sample as a whole (GLAD or UK). For example, 2.8% of the UK population is male aged 16–19, compared to 0.7% of the GLAD population. GLAD participants are represented in light (male) and dark (female) shades of blue, while the UK population is represented in light (male) and dark (female) shades of green.

**Fig. 5:**
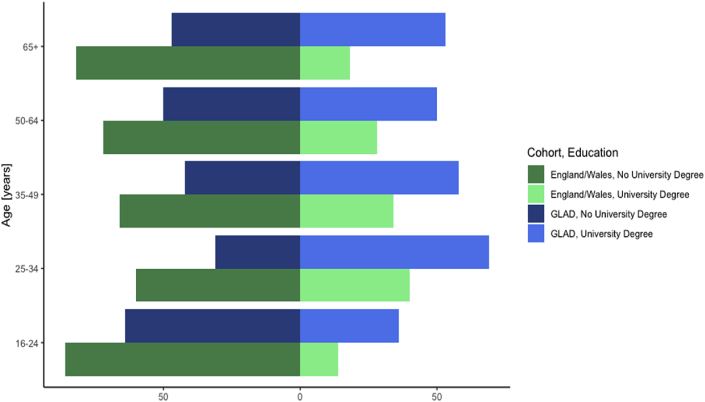
Proportion of individuals with university degrees within age groups in the GLAD sample compared to the English and Welsh population Comparison of education level by age group between the GLAD sample and the general population of England and Wales as measured in the 2011 census (Office for National Statistics, National Records of Scotland, and Northern Ireland Statistics and Research Agency, 2016). Data for Northern Ireland and Scotland was not readily available. Proportions of individuals without university degrees within each age group are displayed on the left side of the graph, with proportions of individuals with university degrees on the right. Percentages represent the proportion within each age group with or without a university degree. For example, 14% of the English population between the ages of 16–24 have a university degree compared to 36% of the GLAD population. GLAD participants are represented in dark (no university degree) and light (university degree) shades of blue, while the English/Welsh population is represented in dark (no university degree) and light (university degree) shades of green.

In the current sample, the mean age is 38.1 with most participants being female, white, and in paid employment or self-employed (Table 2). The GLAD sample is younger and more female (Fig. 4) and has a higher proportion of individuals with a university degree (Fig. 5) than the general UK population. For example, 13.1% of our sample is female aged 25–29 compared to only 4.2% of the UK population. This difference is seen across all age groups under 60. Similarly, in the age-range 25–34 years, 68.9% of the sample have a university degree, whereas in England and Wales this is only 40.4%. In total, 54.8% of the GLAD sample have a university degree compared to 27.2% of the English and Welsh population. Matching educational attainment data was not readily available for Northern Ireland and Scotland.

**Table 2:** Descriptors of GLAD sample, July 2019.

	Percent^a^
Sex	
Male	20.3%
Female	79.7%
Gender	
Transgender	1.5%
Sexuality	
Heterosexual	73.4%
Homosexual	6.5%
Bisexual	15.5%
Asexual	1.3%
Self-define	3.1%
Ethnicity	
White	94.8%
Mixed	2.5%
Asian or Asian British	1.2%
Black or Black British	0.5%
Arab	0.1%
Other	0.9%
Education (highest level achieved)	
College or university degree	54.8%
A levels/AS levels or equivalent	22.7%
O levels/GCSEs or equivalent	14.8%
CSEs or equivalent	1.8%
Employment	
Employed	59.5%
Retired	5.7%
Unable to work because of sickness or disability	11.8%
Unemployed, looking after home/family, or doing voluntary work	8.9%
Full or part-time student	13.0%
Relationship status	
Single	27.8%
Relationship	32.2%
Married/civil partnership	30.3%
Divorced/separated	7.6%
Widowed	1.1%
Smoking status	
Current	17.5%
Former	31.9%
Never	50.6%
Handedness	
Right handed	87.2%
Left handed	11.1%
Ambidextrous	1.7%
Received treatment^b^	
Any treatment	96.1%
Psychological (talking) therapies	81.6%
Medication	89.7%

a Where totals do not sum to 100%, the remaining % was due to incomplete data from those who declined to answer.

b Treatment percentages were only calculated for participants who screened positively for MDD or GAD. Participants were asked to select all that applied in response to the question “Did you ever try or are currently trying the following for these problems?” Participants are considered to have received treatment if they indicated they tried medication prescribed for at least two weeks or tried psychotherapy or other talking therapy more than once.

A high proportion of GLAD participants report the occurrence of at least one form of trauma or abuse throughout their lives (see Table 3). Child abuse was most commonly reported, with emotional abuse by parents or family being endorsed by 42.1% of the sample (in comparison to the estimated 9% of adults in England and Wales who experienced emotional abuse as a child (Office for National Statistics, 2016)). Other forms of life stress are also frequently endorsed with 62.8% of the sample reporting at least one traumatic experience in their lives. Of note, few participants reported periods of inability to pay rent, reflecting low rates of poverty in the sample.

**Table 3:** Proportion of the GLAD sample reporting previous child abuse/neglect, domestic abuse, or traumatic life events.

	Proportion of sample
**Child abuse/neglect**^a^	
Any abuse or neglect	55.4%
Emotional abuse	42.1%
Physical abuse	20.2%
Sexual abuse	20.1%
Emotional neglect	14.8%
Physical neglect	4.2%
**Domestic abuse**^b^	
Any abuse	30.6%
Emotional abuse	37.0%
Physical abuse	19.0%
Sexual abuse	22.6%
**Stressful life event**	
Any stressful/traumatic life event^d^	62.8%
Sexual assault^c^	44.7%
Attacked, mobbed, or robbed^c^	26.7%
Life-threatening accident^c^	13.3%
Witnessed sudden/violent death^c^	15.4%
Life-threatening illness^c^	8.8%
War zone^c^	2.1%
Inability to pay rent	1.6%

a Childhood abuse was assessed by the Childhood Trauma Survey (CTS) (Glaesmer et al., 2013) which includes 5 items on a Likert scale, each relating to the frequency of a distinct type of abuse (emotional, physical, sexual) or neglect (emotional, physical). Percentages reflect the proportion of the sample scoring above the defined cut-off point for clinical utility on each item out of the total.

b Domestic abuse and inability to pay rent was assessed by a separate adult trauma scale without pre-defined cut-offs, therefore cut-off points were devised by the study team based on the CTS scoring. a^,^^b^,^d^Any abuse or stressful/traumatic life event was defined as individuals who scored above the cut-off on at least one item on the respective scale.

c The remaining stressful life events were assessed by combining the sum total of participants indicating “Yes, in the past 12 months” or “Yes, but not in the past 12 months” for each item.

d Any stressful/traumatic life event was defined as individuals who scored above the cut-off point for inability to pay rent or indicated they had experienced one of the stressful/traumatic life events either in the past 12 months or in their lifetime.

### 3.2 Psychopathology

**Fig. 6:**
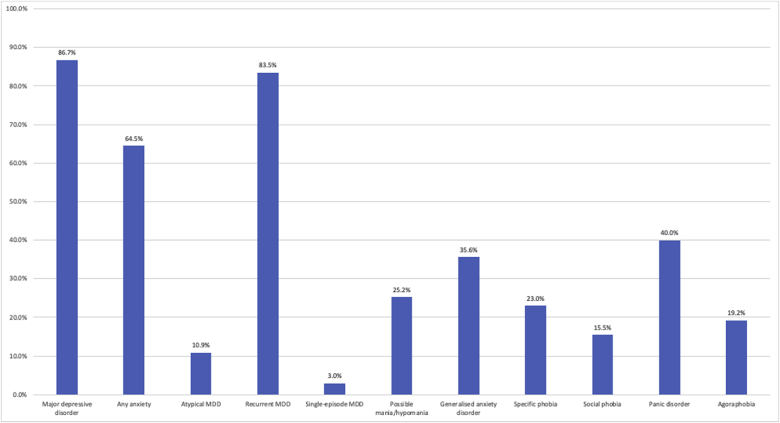
Lifetime prevalence of probable psychiatric disorders in the GLAD sample. Percentages refer to the proportion of participants who meet cut-off criteria on lifetime measures for the specified disorder as defined by the DSM-5 (American Psychiatric Association, 2013) out of the total. Possible mania was assessed by the Mood Disorder Questionnaire (MDQ) (Hirschfeld, 2002).

As shown in Fig. 6, the majority of participants reached diagnostic criteria for major depressive disorder, followed by panic disorder and generalised anxiety disorder. The majority of participants with depression reported recurrent episodes across the lifespan. A figure demonstrating self-reported clinician-provided diagnoses of mental health disorders (as opposed to questionnaire-assessed) in the GLAD sample can be found in supplementary materials (SM 3). Of note, self-reported clinician-provided diagnoses of GAD (77.2%) are twice as high as cases assessed by the questionnaire. The sample also has high rates of comorbidity, with 67.0% of screened MDD cases also screening positively for at least one of the anxiety disorders (GAD, specific phobia, social phobia, panic disorder, or agoraphobia), and 95.1% of screened GAD cases also screening positively for MDD.

Retrospective reports of age of onset indicate a young average onset for the lifetime measures of MDD, GAD, specific phobia, social phobia, panic disorder, and agoraphobia (Table 4). Average age of onset across the anxiety disorders was ∼15. Age of onset for GAD was added to the questionnaire in May 2019, therefore the descriptives below are based on responses from a subset of participants recruited after that date.

**Table 4:** Age of onset in GLAD sample for major depressive disorder, specific phobias, social phobia, panic disorder, and agoraphobia.

	Median	Mean	SD
Major depressive disorder	16	18.61	8.98
Generalised anxiety disorder	16	19.37	11.87
Specific phobia	10	11.32	8.40
Social phobia	12	13.79	8.64
Panic disorder	16	18.80	10.21
Agoraphobia	15	16.90	10.10

Measures of current symptoms of psychopathology included in the questionnaire demonstrate high rates of current clinical presentation of MDD and GAD, with high levels of functional impairment (Table 5).

**Table 5:** Current symptoms of psychopathology and impairment in the GLAD sample.

	Mean	Range	SD	Proportion Clinical Severity	
Major depressive disorder^a^	11.00	0–27	6.92	Clinical (total)	83.7%
				Mild (5–9)	24.7%
				Moderate (10–14)	23.7%
				Moderately severe (15–19)	18.7%
				Severe (20+)	16.7%
Generalised anxiety disorder^b^	9.43	0–21	6.02	Clinical (total)	75.5%
				Mild (5–9)	30.7%
				Moderate (10–14)	21.1%
				Severe (15+)	23.9%
Post-traumatic stress disorder^c^	15.87	0–30	6.29	Clinical (14+)	60.2%
Personality disorder^d^	8.30	0–25	3.65	Clinical (total)	55.0%
				Mild (8–9)	21.9%
				Moderate (10+)	33.1%
Alcohol use^e^	7.06	0–40	6.48	At-risk (total)	38.7%
				Increasing risk (8–15)	27.0%
				Higher risk (16–19)	5.4%
				Possible dependence (20+)	6.4%
Functional Impairment^f^	16.99	0–40	9.07	Clinical (total)	77.8%
				Significant impairment (10–20)	43.5%
				Severe impairment (21+)	34.4%

Percentages refer to the proportion of participants out of the total sample who meet cut-off criteria on current measures for the specified disorder.

a Criteria met for major depressive disorder on PHQ-9 (Kroenke et al., 2001).

b Criteria met for generalised anxiety disorder on GAD-7 (Spitzer et al., 2006).

c Criteria met for PTSD on PCL-6 (Lang et al., 2012) in the past month.

d Criteria met for a personality disorder on SASPD (Olajide et al., 2018).

e Criteria met for at-risk alcohol dependence on AUDIT (Saunders et al., 1993) in the past year.

f Criteria met for functional impairment on WSAS (Mundt et al., 2002).

### 3.3 Media campaign

In order to assess the success of the media campaign, participants were asked to report how they found out about the GLAD Study at the end of the sign-up questionnaire. Results of the campaign were assessed 3 months after the initial launch in England. Participants who completed the questionnaire reported that the three most common ways of hearing about the study were through Facebook, print newspaper, and Twitter (SM 4).

**Fig. 7:**
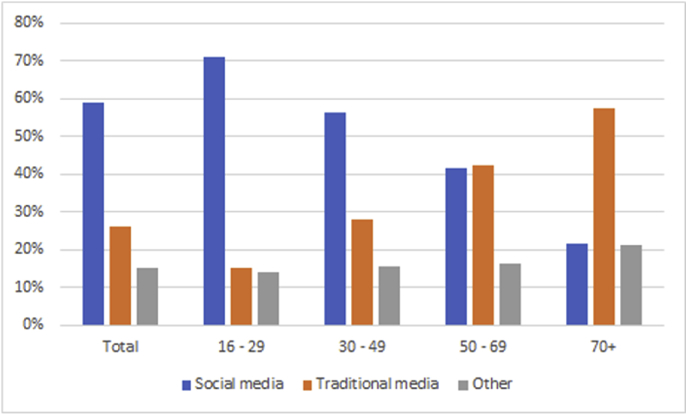
Media campaign drivers to complete questionnaires, by age group. Data is based on responses from 12,714 participants recruited in England between September–December 2019 to assess the success of the launch campaign. Participants were prompted to select all relevant responses to the question “*How did you hear about the GLAD Study?*” Social media refers to Facebook, Twitter, Instagram, bloggers, and search engines. Traditional media refers to radio, TV, newspaper, and online tabloids. All remaining responses are included in the Other category.

The effectiveness of each recruitment strategy varied by age (see Fig. 7). Of participants aged 16–29, the majority learned about the study through social media (Facebook, Twitter, Instagram, search engine), with only 12% receiving study information through traditional media (TV, radio, print newspaper, online tabloid). Participants aged 30–49 also primarily learned about the study through social media, but 22% within that range learned about the study through traditional media. Social media was less effective in reaching individuals aged 50+, with traditional media being the primary means of recruitment above that age.

## 4 Discussion

The GLAD Study represents a large and comprehensive study of anxiety and depression and a valuable resource for future research. By achieving our goal of recruiting 40,000 participants with a lifetime occurrence of one of these disorders into the NIHR Mental Health BioResource, the study will collect detailed, homogenous phenotype and genotype data, thereby increasing power for genetic analyses. Importantly, the recontactable nature of GLAD means that researchers will be able to conduct new studies integrating psychological and genetic data.

Why integrate genetic data? GLAD provides researchers with a way of investigating polygenic and environmental effects in studies with participants. As such, the study is not merely a way to increase GWAS sample size, but also a way to increase the potential utility of genomics within psychological studies of all kinds. Previous efforts in depression and anxiety genetics have been hampered by the unreliable methodology of candidate gene studies (Border et al., 2019). However, Border also concluded that modern and successful genome-wide studies of depression, rigorously testing millions of genetic variants in large samples (e.g. CONVERGE consortium, 2015; Wray et al., 2018), provide reliable evidence. The implications that these highly complex, but highly replicable, polygenic effects have for the field need to be investigated (Hyman, 2018). GLAD exists to enable recruitment of participants into studies not only on the basis of their self-reported information and/or clinical records, but also on the basis of the increasingly well powered method of polygenic risk scoring. This approach combines risk alleles across the genome into a single quantitative measure for any one individual (Belsky and Harden, 2019). For instance, a clinical trial could be undertaken with selection of participants based on past medical history and/or polygenic scores for a relevant trait.

External researchers will be able to apply for access to anonymised data and will have the opportunity to recontact participants for additional data collection. This offers the prospect of a wide range of future recall studies, including clinical trials, observational studies, neuroimaging, and experimental medicine. Recruitment for recall studies could also be targeted to participants with phenotypes or genotypes of interest by utilising GLAD data to screen for specific eligibility criteria, thereby simplifying and expediting the recruitment process for future studies. Furthermore, measures were in part selected to match other cohorts and facilitate meta-analysis with samples such as the UK Biobank, Generation Scotland, and the Australian Genetics of Depression Study, although demographic differences between the samples would need to be taken into account (e.g. age differences between GLAD and UK Biobank participants). We propose that the GLAD data could be useful for stratifying cases into distinct phenotypes of depression and anxiety to reduce heterogeneity, a strategy which has been shown to increase the power of genetic analyses to detect significant effects (CONVERGE consortium, 2015; Power et al., 2017; Verduijn et al., 2017).

Initial descriptive analyses reveal that the current GLAD sample does not reflect the demographics of the general population of the UK. The limited ethnic diversity within the sample is not representative, with 95% of participants being white compared to 87% of the population (Office for National Statistics, National Records of Scotland, and Northern Ireland Statistics and Research Agency, 2016). Similarly, although common mental disorders are more than twice as common in young women compared to young men, and 1.5 times more common in women overall (NHS Digital, 2016), the GLAD sample remains disproportionately female. Finally, roughly double the proportion of the study sample report having a university degree compared to the English and Welsh population.

The GLAD sample as a whole also shows severe psychopathology. Our lifetime questionnaire diagnostic algorithm indicated that the majority of the sample have recurrent depression, and 20.9% report possible mania/hypomania. Over half the sample screen positively for a lifetime anxiety disorder, with panic disorder being the most common. Participants also show substantial comorbidity, in particular for GAD cases reporting a concurrent diagnosis of MDD. Our results show significantly higher comorbidity rates for GAD cases compared to a previous epidemiological study whereby 62.4% of GAD cases had a lifetime occurrence of MDD (Judd et al., 1998). However, this is likely further indication of the sample's severity, reflecting previous reports of higher rates of comorbid anxiety in patients with recurrent lifetime depression (Boland, Keller, Gotlib, & Hammen, 2002; Moffitt et al., 2007). In terms of current symptoms, GLAD participants report moderate symptoms of depression, mild to moderate symptoms of anxiety, and significant functional impairment, indicating poor work and social adjustment. Finally, it was interesting to note that 55.0% of participants screened positive for mild-moderate personality disorder.

Severity levels are also demonstrated by the 96.1% of GLAD participants who report receiving treatment for depression or anxiety, although the study is open to individuals regardless of treatment receipt. In Western countries, it is estimated that between one-thirds and one-half of patients with depression or anxiety disorders do not receive a diagnosis and/or treatment (Kessler, Bennewith, Lewis, & Sharp, 2002), a group not represented in our sample. However, even in those patients receiving treatment, recent research suggests that only 20% get minimally adequate treatment (Thornicroft et al., 2017), meaning that follow-up research on treatment efficacy in the GLAD sample would be highly valuable.

An unanticipated benefit of the campaign was the interest it generated from UK NHS sites around the country. Over 100 NHS sites, including Trusts and GP practices, contacted the study team with interest in supporting recruitment, and many had learned about the study from the media campaign. The study was also adopted onto the NIHR Clinical Research Network (CRN) Portfolio which provides support and funding for NHS organisations that are involved in research. The combined approach of both general and clinical recruitment reaches a wider demographic of participants than either strategy alone. Patients recruited through clinics can also be assisted in signing up by a local clinician or research team to provide support throughout the process if needed.

In addition to collecting a large amount of data, the GLAD study represents a template for future online studies. The media campaign proved highly effective in recruiting a large number of participants in a short amount of time and demonstrates the success of such a broad outreach approach. Caution must be taken when interpreting the success of the different media campaign strategies, given the prolonged use of social media promotions in comparison to traditional media outlets; nonetheless social media was the most effective strategy for recruiting individuals under 50. Recruiting younger participants not only reflects the young average age of onset of anxiety and depression, approximately 11 (Bandelow & Michaelis, 2015) and 24 (Christie et al., 1988; Kessler et al., 2003) respectively, but also helps to facilitate long-term follow up and recontact.

### 4.1 Limitations

As previously mentioned, the current demographics of study participants who completed the questionnaire are not representative of the population of the UK. This suggests a selection bias comparable to what has been observed in similar studies such as the UK BioBank (Fry et al., 2017; Adams et al., 2019). Results from analyses of the current GLAD sample therefore may not be generalisable to the whole population. Smaller studies could overcome these biases by selectively over-sampling males and individuals at the lower end of educational attainment to be more representative of the population.

However, efforts are being taken to recruit a wider demographic of participants into the study. Specifically, we will develop a targeted social media campaign to reach young men and collaborate with young male influencers to appeal to that audience. We will additionally prepare a future media campaign that focuses on depression and anxiety separately, with wording specific to each disorder, to attempt to reduce the high comorbidity rates in the sample. Furthermore, we are collaborating with local branches of the charity Mind. These are typically placed within the community and provide a range of mental health support services. Branches will be displaying posters and leaflets on site, posting on social media, and answering questions or providing assistance to potential participants interested in signing up. Recruitment through NHS services and the availability of local research teams within those sites will also help reach the general population and recruit individuals with a wider range of educational attainment.

Of particular importance, we are working with Black, Asian, and minority ethnic charities and influencers to conduct additional user testing to understand the barriers to participating for non-white individuals and increase outreach to diverse communities. Previous genetic studies have involved primarily individuals of white European ancestry (Haga, 2010), and findings from these studies may not apply to individuals of non-white descent. By actively recruiting a diverse sample, our objective is to additionally facilitate research on typically underrepresented groups.

Another challenge of the study design is the drop-out rates following recruitment. At each stage of the sign-up process, a substantial number of participants did not complete the next step despite multiple reminders. Unfortunately, we are unable to assess the demographics of participants who do not complete the sign-up questionnaire, but it is possible that the drop-outs are non-random and represent a certain phenotype, such as individuals with more severe symptoms or lower health literacy.

## 5 Conclusion

The GLAD Study offers a recontactable resource of participants with a lifetime occurrence of depression and anxiety disorders to facilitate future health research. The online study design and media recruitment strategy were effective in recruiting a large number of individuals with these disorders into the NIHR Mental Health BioResource. Recruitment is ongoing with the goal of completing recruitment of 40,000 individuals, making this the largest single study of depression and anxiety. We hope that this paper will not only demonstrate the effectiveness of the study methodology but will also raise awareness of the availability of this cohort to researchers in the field and promote future collaboration.

## Funding

This work was supported by the National Institute of Health Research (NIHR) BioResource, NIHR Biomedical Research Centre [IS-BRC-1215-20018], HSC R&D Division, Public Health Agency [COM/5516/18], MRC Mental Health Data Pathfinder Award (MC_PC_17,217), and the National Centre for Mental Health funding through Health and Care Research Wales.

## Declaration of competing interest

Prof Breen has received honoraria, research or conference grants and consulting fees from Illumina and Otsuka. Prof Eley and Dr Breen are part-funded by a program grant from the UK Medical Research Council (MR/M021475/1). Prof Cleare has in the last three years received honoraria, travel funding, or consultancy fees from Lundbeck, Livanova, Allergan and Janssen. Prof McIntosh has received research support from Eli Lilly, Janssen, and the Sackler Foundation, and has also received speaker fees from Illumina and Janssen. Prof Walters has received grant funding from Takeda for work unrelated to the GLAD Study.

## Footnotes

2 Professor Chérie Armour, the Principal Investigator (PI) in Northern Ireland (NI) moved institution during the course of the study from Ulster University to Queens University Belfast, remaining as the NI PI.
