# Supplementary material for: The Genetic Links to Anxiety and Depression (GLAD) Study: Online recruitment into the largest recontactable study of depression and anxiety
Source: Behav Res Ther. 2019 Dec;123:103503. doi: 10.1016/j.brat.2019.103503 (PMC6891252; doi:10.1016/j.brat.2019.103503)
Supplement: Multimedia component 1 [file mmc1.docx]

**The Genetic Links to Anxiety and Depression (GLAD) Study: online recruitment into the largest study of depression and anxiety**

**Supplementary Materials**

**SM 1.** Optional modules in the GLAD Study

| **Assessment/Topic** | **Purpose** | **Source** |
| --- | --- | --- |
| **Fear** | Assess severity of fears to a variety of stimuli. | Fear Survey Schedule (Wolpe & Lang, 1969) |
| **Drugs and addictions** | Screening tool for drug-use problems. Assess severity and frequency of addiction-associated behaviours | Drug Use Disorder Identification Test (DUDIT) (Berman, Bergman, Palmstierna, & Schlyter, 2003) and UK Biobank Mental Health Questionnaire (Davis et al., 2018) |
| **Obsessive-compulsive disorder** | Assess severity of obsessive-compulsive symptoms | Obsessive-Compulsive Inventory, shorter version (OCI-R) (Foa et al., 2002) |
| **Post-traumatic stress disorder and trauma** | Screen for post-traumatic stress disorder to make a provisional diagnosis and assesses severity of symptoms. This tool can also be used to monitor symptom change over time. | PTSD Checklist for DSM-5 (PCL-5) (Weathers, Litz, et al., 2013) |
| **Perinatal depression** | Identify those currently at risk of suffering from perinatal depression | Edinburgh Postnatal Depression Scale (EPDS) (Cox, Holden, & Sagovsky, 1987) |
| **Physical appearance** | Screening measure for body dysmorphic disorder to assess the severity of dysmorphic concern. | Dysmorphic Concerns Questionnaire (DCQ) (Mancuso, Knoesen, & Castle, 2010) |
| **Eating disorders** | Assess presence of eating disorders (DSM-5) including anorexia nervosa, atypical anorexia nervosa, bulimia nervosa, binge-eating disorder, purging disorder and night eating syndrome. Can also assess the severity of muscle dysmorphia in men. | Adapted from the ED100K (Thornton et al., 2018) (to include additional bespoke atypical anorexia nervosa questions written by the study team), BEDS-7 (Herman et al., 2016), NEQ (Allison, Stunkard, & Thier, 2004) and MDDI (Hildebrandt, Langenbucher, & Schlundt, 2004). |
| **Vomit phobia** | Assess severity of vomit phobia | Specific Phobia of Vomiting Inventory (SPOVI) (Veale et al., 2013) |
| **Experiences of healthcare** | Assess recent experience with health services in the UK | Adapted from the AU Genetics of Depression questionnaire (https://www.geneticsofdepression.org.au/) |
| **Life events** | Assess lifetime experience of life events or situations | Adapted from the AU Genetics of Depression questionnaire, includes the Life Events Checklist for DSM-5 (LEC-5) (Weathers, Blake, et al., 2013) |
| **Work and sleep** | Assess general work and sleep pattern | Adapted from the AU Genetics of Depression questionnaire |
| **Games and gambling** | Identify experiences of various kinds of gambling. This includes placing a bet on the outcome of a race or a game of skill or chance, or playing a game, including for charity, in which you might win or lose your money. | Adapted from the AU Genetics of Depression questionnaire |
| **Headaches and migraines** | Assess experiences, severity and treatments used for headaches and/or migraines | Adapted from the AU Genetics of Depression questionnaire |
| **Family history** | Gathers basic demographical information on members of immediate family and identifies any possible hereditary illnesses or disorders | Adapted from the AU Genetics of Depression questionnaire, the NIHR Mental Health BioResource common subject assessment, and the NIHR BioResource general health and lifestyle questionnaire |
| **Medication** | Gathers information on any medication taken predominantly to treat a mental health disorder, including, but not limited to, anxiety and depression | Adapted from the AU Genetics of Depression questionnaire by the study team based on common medications taken in the UK for mental health disorders |

**SM 2.** Data and sample access request protocol

*Recall Study Processes*

The GLAD Study is part of the NIHR BioResource which has been established to support recruitment of participants to research studies according to their physical characteristics (phenotype) and/or genetic makeup (genotype) where this would not have been effective/possible using more general methods of participant recruitment (e.g. posters and general advertising). Analysis of DNA samples to detect individual base pair variants where one 'letter' in the genetic code is different (single nucleotide polymorphism) and screening for variations across the genome (array based genotyping or sequencing the coding parts of the genome (exome sequencing) or the whole genome (whole genome sequencing)) will allow individuals to be recalled for research studies according to their genetic makeup. Analysis of serum, plasma or cells for biomarkers and information provided by participants and held in the database will allow individuals to be recalled according to physical characteristics and health and lifestyle habits. This will allow participants who match study criteria and who have consented to be contacted about research studies to be approached about specific studies. The maximum number of invitations to studies will be four in every 12 months

Applications to undertake recall studies involving GLAD Study participants or access to samples or data are reviewed by the Scientific Advisory Board of the NIHR Mental Health BioResource (for studies recruiting from GLAD) or the Steering Committee of the NIHR BioResource (for external studies). Details of the application process can be obtained by contacting gladstudy@kcl.ac.uk.

Studies involving recall of participants through the GLAD Study or NIHR BioResource require separate approval by a Research Ethics Committee and the Health Research Authority. Once this and any other regulatory approvals (for example Human Tissue Authority, Medicines and Health Regulatory Authority) are in place the recall of participants is coordinated and handled by the GLAD Study or NIHR BioResource team, either locally or through the national coordinating centre. For some studies, for example where a fresh blood sample is required, the GLAD Study or NIHR BioResource team may also be responsible for consenting participants and collection of samples.

The GLAD Study and NIHR BioResource receives funding from the National Institute for Health Research. It does not generate any profit, but to cover operational costs a service fee may be charged. Industry can access the resource on a cost recovery basis; details of charges are available at https://bioresource.nihr.ac.uk/.

A condition of use of the GLAD Study or NIHR BioResource will be the feedback of research outcomes to the participants. It will be the responsibility of the research team to produce feedback information and the BioResource will be responsible for mailing or communicating the pre-prepared lay summary of the study outcomes.

*Management of sample and/or data access requests*

Requests from researchers for access to existing samples and/or data will be assessed by the Steering Committee or the Scientific Advisory Board of the GLAD Study, NIHR Cambridge BioResource, or NIHR BioResource. Release of any samples and/or data will be covered by a Material/Data Transfer (MTA/DTA) agreement with all individuals/organisations requesting access, whether they reside within or outside the UK. The MTA/DTA is a legally binding document that will regulate the use of samples and data to ensure that standards are maintained.

Where samples are released and there is a surplus after the study is completed the return, disposal or storage will be determined by the arrangements described in the study specific protocol and Research Ethics application. If samples are released through the generic ethical approval of the Research Tissue Bank the arrangements will be described in the Material Transfer Agreement. To avoid unauthorised use of samples outside the study, researchers are required state the time period for the study, and a review time point will be agreed within the MTA.

A condition of access to samples is that all research data generated should be deposited in a GLAD Study or an NIHR BioResource approved depository. This will enhance cross-interrogation of different datasets, enable secure biomedical analysis and facilitate the recall of participants for further studies.

**SM 3.** Rates of self-reported clinician-provided diagnoses of mental health disorders

**Participants were asked to select all relevant diagnoses, prompted by the question: “Have you ever been diagnosed with one or more of the following mental health problems by a professional, even if you don't have it currently? By professional we mean: any doctor, nurse or person with specialist training (such as a psychologist, psychiatrist etc.). Please include disorders even if you did not need treatment for them or if you did not agree with the diagnosis.”*

**SM 4.** Referral rates to the GLAD Study by age

| Age | | | | | | | | | | |
| --- | --- | --- | --- | --- | --- | --- | --- | --- | --- | --- |
|  | **16 - 29** | | **30 - 49** | | **50 - 69** | | **70+** | | **Total** | |
|  | N | % | N | % | N | % | N | % | N | % |
| Facebook | 2693 | 51.1% | 2374 | 41.5% | 673 | 28.0% | 35 | 14.8% | 5775 | 42.3% |
| Twitter | 556 | 10.5% | 690 | 12.1% | 261 | 10.8% | 4 | 1.7% | 1511 | 11.1% |
| Instagram | 389 | 7.4% | 53 | 0.9% | 0 | 0.0% | 0 | 0.0% | 442 | 3.2% |
| Blogger | 51 | 1.0% | 11 | 0.2% | 5 | 0.2% | 1 | 0.4% | 68 | 0.5% |
| Search engine | 63 | 1.2% | 94 | 1.6% | 57 | 2.4% | 11 | 4.6% | 225 | 1.7% |
| Radio | 31 | 0.6% | 113 | 2.0% | 106 | 4.4% | 13 | 5.5% | 263 | 1.9% |
| TV | 138 | 2.6% | 223 | 3.9% | 213 | 8.9% | 27 | 11.4% | 601 | 4.4% |
| Newspaper | 409 | 7.8% | 937 | 16.4% | 545 | 22.6% | 80 | 33.8% | 1971 | 14.5% |
| Online tabloid | 215 | 4.1% | 333 | 5.8% | 152 | 6.3% | 16 | 6.8% | 716 | 5.1% |
| Word of mouth | 430 | 8.2% | 405 | 7.1% | 142 | 5.9% | 19 | 8.0% | 996 | 7.3% |
| Employer | 89 | 1.7% | 81 | 1.4% | 37 | 1.5% | 2 | 0.8% | 209 | 1.5% |
| Charity | 97 | 1.8% | 195 | 3.4% | 69 | 2.9% | 9 | 3.8% | 370 | 2.7% |
| Clinician/GP | 7 | 0.13% | 9 | 0.16% | 6 | 0.25% | 0 | 0.00% | 22 | 0.16% |
| Other | 105 | 1.99% | 208 | 3.63% | 141 | 5.86% | 20 | 8.44% | 474 | 3.47% |
| Total Responders | 5273 | 100.0% | 5726 | 100.0% | 2407 | 100.0% | 237 | 100.0% | 13643 | 100.0% |

Participants were prompted to select all relevant responses to the question “*How did you hear about the GLAD Study?*” Social media includes Facebook, Twitter, Instagram, bloggers, and search engines. Traditional media includes radio, TV, newspaper, and online tabloids.

Bibliography

Allison, K. C., Stunkard, A. J., & Thier, S. L. (2004). *Overcoming night eating syndrome: a step-by-step guide to breaking the cycle*. New Harbinger Publications.

Australian Genetics of Depression study – Genetics of depression. (n.d.). Retrieved January 31, 2019, from https://www.geneticsofdepression.org.au/

Berman, A., Bergman, H., Palmstierna, T., & Schlyter, F. (2003). The Drug Use Disorders Identification Test (DUDIT) Manual. *Stockholm, Sweden: Karolinska Institutet*.

Cox, J. L., Holden, J. M., & Sagovsky, R. (1987). Detection of postnatal depression. Development of the 10-item Edinburgh Postnatal Depression Scale. *The British Journal of Psychiatry*, *150*, 782–786.

Davis, K. A. S., Coleman, J. R. I., Adams, M., Allen, N., Breen, G., Cullen, B., … Hotopf, M. (2018). Mental health in UK Biobank: development, implementation and results from an online questionnaire completed by 157 366 participants. *BJPsych Open*, *4*(3), 83–90. doi:10.1192/bjo.2018.12

Foa, E. B., Huppert, J. D., Leiberg, S., Langner, R., Kichic, R., Hajcak, G., & Salkovskis, P. M. (2002). The Obsessive-Compulsive Inventory: Development and validation of a short version. *Psychological Assessment*, *14*(4), 485–496. doi:10.1037/1040-3590.14.4.485

Herman, B. K., Deal, L. S., DiBenedetti, D. B., Nelson, L., Fehnel, S. E., & Brown, T. M. (2016). Development of the 7-Item Binge-Eating Disorder Screener (BEDS-7). *The Primary Care Companion for CNS Disorders*, *18*(2). doi:10.4088/PCC.15m01896

Hildebrandt, T., Langenbucher, J., & Schlundt, D. G. (2004). Muscularity concerns among men: development of attitudinal and perceptual measures. *Body Image*, *1*(2), 169–181. doi:10.1016/j.bodyim.2004.01.001

Mancuso, S. G., Knoesen, N. P., & Castle, D. J. (2010). The Dysmorphic Concern Questionnaire: A screening measure for body dysmorphic disorder. *The Australian and New Zealand Journal of Psychiatry*, *44*(6), 535–542. doi:10.3109/00048671003596055

Thornton, L. M., Munn-Chernoff, M. A., Baker, J. H., Juréus, A., Parker, R., Henders, A. K., … Bulik, C. M. (2018). The Anorexia Nervosa Genetics Initiative (ANGI): Overview and methods. *Contemporary Clinical Trials*, *74*, 61–69. doi:10.1016/j.cct.2018.09.015

Veale, D., Ellison, N., Boschen, M. J., Costa, A., Whelan, C., Muccio, F., & Henry, K. (2013). Development of an inventory to measure specific phobia of vomiting (emetophobia). *Cognitive Therapy and Research*, *37*(3), 595–604. doi:10.1007/s10608-012-9495-y

Weathers, F. W., Blake, D. D., Schnurr, P. P., Kaloupek, D. G., Marx, B. P., & Keane, T. M. (2013). The Life Events Checklist for DSM-5 (LEC-5). *Instrument Available from the National Center for PTSD at Www. Ptsd. Va. Gov*.

Weathers, F. W., Litz, B. T., Keane, T. M., Palmieri, P. A., Marx, B. P., & Schnurr, P. P. (2013). The PTSD Checklist for DSM-5 (PCL-5). *National Center for PTSD*.

Wolpe, J., & Lang, P. J. (1969). *Fear Survey Schedule*. San Diego, California: Educational and Industrial Testing Service.
